# Concomitant effect of high altitude and thrombophilia on multi-organ thrombosis

**DOI:** 10.21542/gcsp.2026.22

**Published:** 2026-06-30

**Authors:** Feridoun Sabzi, Reza Faraji, Fariba Shokri, Abbas Ghaysouri, Abbas Maleki, Taha Rashidi

**Affiliations:** 1 Department of General Surgery, School of Medicine, Kermanshah University of Medical Sciences, Kermanshah, Iran; 2 Tuberculosis and Lung Diseases Research Center, Ilam University of Medical Sciences, Ilam, Iran; 3 Clinical Microbiology Research Center, Ilam University of Medical Sciences, Ilam, Iran; 4 Student Research Committee, Kermanshah University of Medical Sciences, Kermanshah, Iran

## Abstract

We report the case of a 33-year-old male mountaineer, presenting with chest pain, severe headache, dyspnea, and lower extremity edema, on his first expedition to Paraw mountain located in Kermanshah Province. His condition involved a complex thrombotic presentation, including both venous and arterial events, such as a myocardial infarction due to stenosis of the left main coronary artery, which we believe to be a coincidental pathology. His neurological symptom includes headache, vomiting, and slight drowsiness associated with chest pain, which were evaluated alongside confirmatory testing for protein C-S deficiency. Physical examination revealed right lower extremity edema, respiratory distress (RR = 34), and mid-drowsiness. Chest X ray showed lung congestion. A history of recurrent thrombophlebitis, along with the current myocardial infarction, raised suspicion of a thrombophilic state. The final diagnosis of these complications was confirmed to have protein C-S deficiency based on laboratory tests performed during the acute phase. However, the hereditary nature of this deficiency remains unconfirmed as no genetic testing or family screening was undertaken. The patient underwent coronary artery bypass grafting of left anterior descending artery (LAD) and LCX associated with anticoagulant therapy, and his neurologic sign and symptom and lower extremity deep vein thrombosis recovered uneventfully.

## Introduction

Protein C is a vitamin K–dependent plasma protein with both anticoagulant and profibrinolytic properties^1^. It is activated by thrombin bound to thrombomodulin on the endothelial surface; activated protein C then inactivates factors Va and VIIIa, thereby downregulating thrombin generation. Protein S serves as a cofactor for these actions of protein C^2^. Deficiency of protein C or protein S is an inherited trait that predisposes to venous thromboembolism^3^.

Cerebral venous sinus thrombosis, including thrombosis of the superior sagittal sinus, has a broad range of aetiologies, most notably thrombophilic states such as factor V Leiden mutation, elevated anticardiolipin antibodies, antithrombin deficiency, protein C and protein S deficiencies, hyperhomocysteinaemia, and polycythaemia vera^4^.

We report a patient with combined protein C and protein S deficiency who presented with concurrent peripheral thrombophlebitis, superior sagittal sinus thrombosis, and myocardial infarction. Whether these three events share a single thrombophilic mechanism or represent a coincidental co-occurrence cannot be established with certainty from a single case. Cerebral venous sinus thrombosis (CVST) is an uncommon and frequently under-recognised form of stroke, with an estimated incidence of approximately five per million per year and accounting for 0.5–1% of all strokes; when associated with an underlying thrombophilia, it most often affects young adults.

## Case presentation

A 33-year-old male, with a history of two previous episodes of lower extremity thrombophlebitis, went on expedition to the Paraw mountain (3,400 m) in Kermanshah Province for recreation.

On day two of his expedition near the summit, he developed neurological dysfunction in the form of severe headache, motor weakness, acute dyspnea, and left leg edema. On day three, he was evacuated to the nearest hospitals by his friends to receive emergency medical care (Lasix and hyperbaric oxygen therapy) and then brought to our hospital.

Physical examination revealed a pulse of 120 beats/min and a blood pressure of 80/40 mmHg. The patient was in cardiogenic shock and had tachypnea and diffuse inspiratory crepitating rales.

Transthoracic echocardiography showed anterolateral hypokinesis with an ejection fraction of 30% and mild mitral regurgitation. The ECG revealed sinus rhythm, anteroseptal Q waves, and the chest X-ray showed infiltrates consistent with acute pulmonary edema.

Results of initial tests of cardiac enzymes indicated the myocardial band (MB) level of 165 U/L, troponin level of 2 ng/ml (normal level: <0.04 ng/ml), and creatine phosphokinase level of 2,133 u/l.

**Figure 1.**
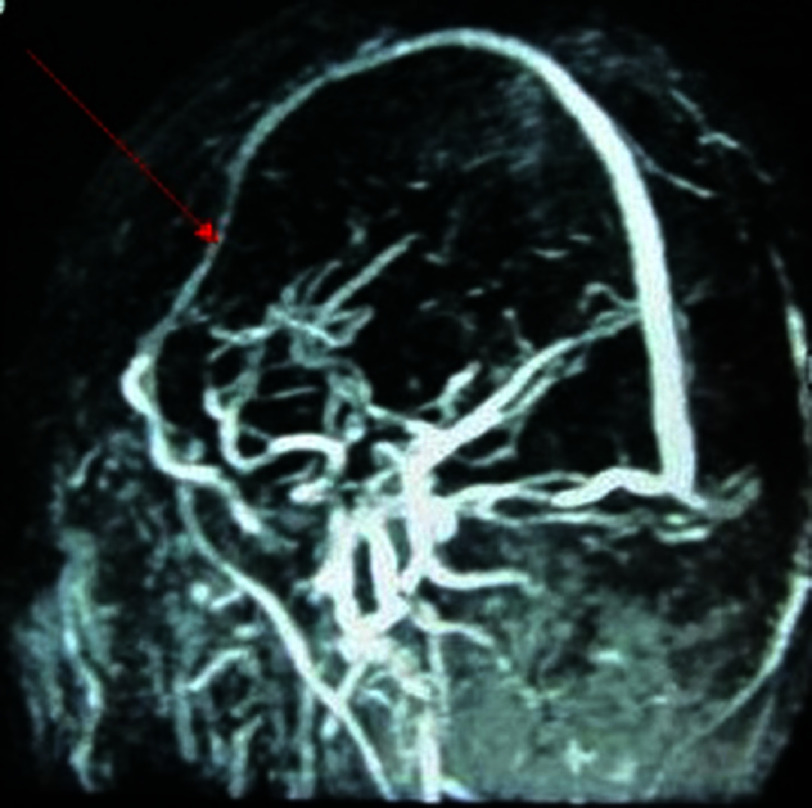
MR venography demonstrating thrombosis of the superior sagittal sinus.

**Figure 2.**
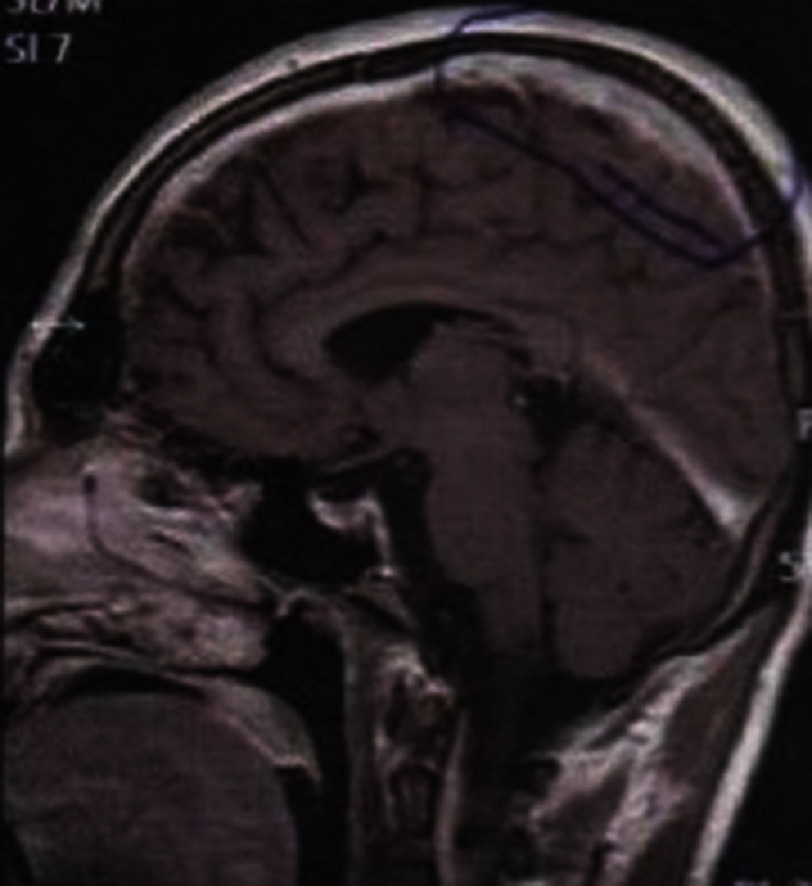
Contrast-enhanced MRI demonstrating thrombosis of the superior sagittal sinus.

A recurrent history of thrombophlebitis, along with the current myocardial infarction and neurological symptoms, raised suspicion of a thrombophilic state. Progressive positional headache suggested that magnetic resonance imaging (MRI) should be performed. MRI venography and brain angiography revealed sagittal vein thrombosis (Figures 1 and 2), and laboratory examination confirmed protein C-S deficiency.

**Figure 3.**
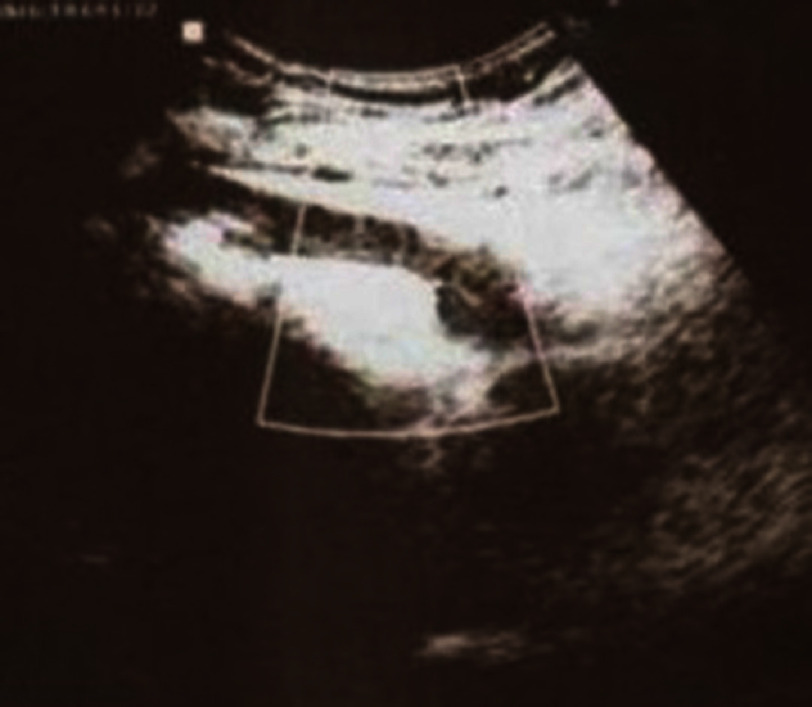
Doppler ultrasound showing thrombotic and non-compressible saphenous vein.

**Figure 4.**
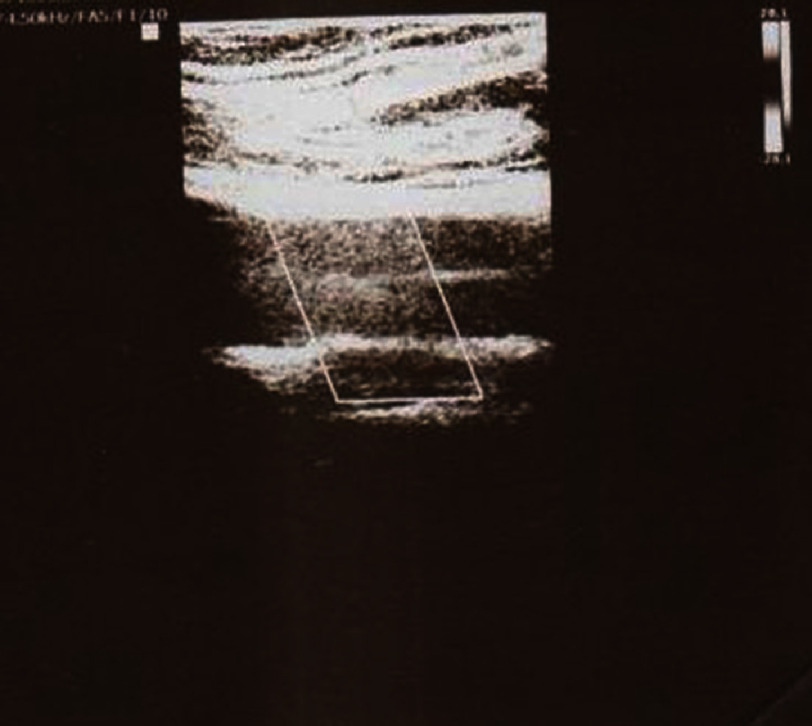
Doppler ultrasound showing non-compressible saphenous vein due to thrombosis.

Doppler ultrasound of the lower extremities, conducted pre-operatively, revealed thrombosis in the left proximal superficial femoral vein and left popliteal vein extending into the posterior tibial vein (Figures 3,  4). Total blood count and blood biochemistry assay were within the normal limits; however, the erythrocyte sedimentation rate was prolonged, and protein C and S levels reduced. During the acute event, the protein C level was 53% (normal: >70%), and protein S level was 50% (normal: 65%), which may be transiently depressed due to the acute-phase response and anticoagulation therapy.

In the acute phase of the patient’s illness, protein C and S levels were measured, but confirmatory genetic testing and family screening were not conducted. The diagnosis of protein C-S deficiency was based on the clinical presentation, laboratory findings, and prior history of thrombophlebitis. Future studies could include genetic testing and family screening to definitively confirm the hereditary nature of this deficiency.

Lupus anticoagulant, antinuclear antibody, anti–double-stranded DNA antibody, anticardiolipin antibodies, antithrombin III, rheumatoid factor, HLA-B27, and HLA-B5 were all within normal limits or negative. The patient developed progressive respiratory failure and hypotension, requiring mechanical ventilation. Despite high-dose adrenaline and dobutamine, intra-aortic balloon pump counterpulsation was required to stabilise the haemodynamic state.

**Figure 5.**
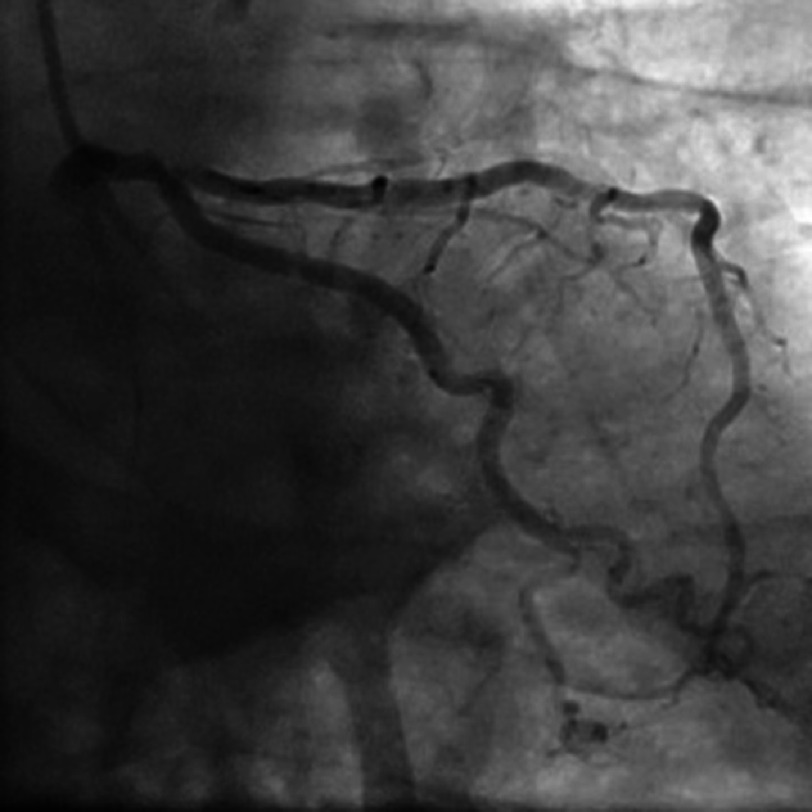
Coronary angiography demonstrating severe stenosis of the left main coronary artery.

Coronary angiography demonstrated significant stenosis of the left main coronary artery, with a normal right coronary artery (Figure 5). The pulmonary artery systolic pressure was 80 mmHg. The patient’s subsequent course was complicated by acute renal failure. Given the haemodynamic instability associated with the acute myocardial infarction, immediate surgical revascularisation was undertaken. Off-pump coronary artery bypass grafting was performed *via* median sternotomy, with the left internal mammary artery grafted to the left anterior descending artery and a saphenous vein graft to the left circumflex artery.

The patient underwent peritoneal dialysis immediately on transfer to the intensive care unit. Postoperative recovery was slow; the intra-aortic balloon pump and inotropic support were withdrawn on the seventh postoperative day. The neurological dysfunction and acute renal failure resolved over 22 days, and the patient was discharged on the thirtieth postoperative day on oral warfarin.

## Discussion

Vascular thrombosis is an uncommon but recognised complication of high-altitude exposure in predisposed individuals. Prolonged exposure to high altitude, particularly in those with an underlying genetic thrombophilia, can increase thrombotic risk through dehydration, haemoconcentration, and reduced physical activity, all of which promote a hypercoagulable state.

Several factors contribute to hypercoagulability at high altitude^4^. In the present case, as described by Maher et al.^5^, the principal contributors include reduced physical activity, coagulation disorders, and severe dehydration. Genton et al.^6^ reported that enhanced coagulation may also arise from abnormal platelets with increased adhesiveness, red-cell anisocytosis, and polycythaemia, producing microthrombi that initiate the coagulation cascade.

The role of microthrombi in acute mountain sickness has been described theoretically by Huisman et al. and Fujimaki et al.,^7,8^ underscoring the contribution of high altitude to hypercoagulability. More recent studies suggest that high-altitude exposure can produce abnormal coronary vasomotor responses and may increase the risk of cardiovascular events, including myocardial infarction, particularly in individuals with pre-existing cardiovascular disease^9^.

However, Bärtsch et al.^10^ found no evidence that, in healthy individuals, altitude exposure produces the haematological or coagulation changes that would increase the risk of plaque rupture, vascular thrombosis, or myocardial infarction.

We propose that altitude-induced endothelial changes, in combination with protein C and protein S deficiency, predisposed this patient to vasomotor abnormality, thrombosis, and myocardial infarction. Specifically, we postulate that ascent to high altitude acted as an additional prothrombotic stimulus superimposed on the congenital protein C and S deficiency, contributing to the cerebral venous thrombosis. In cases clinically suggestive of isolated cerebral venous thrombosis, brain CT should serve as the initial diagnostic investigation, prompting MRI when further assessment is required.

## What have we learnt?

• In this patient, combined protein C and protein S deficiency—an inherited thrombophilia—together with high-altitude exposure (3,421 m) was associated with multi-organ venous thrombosis involving the cerebral, cardiac, pulmonary, and lower-limb circulations.
• Contributing factors at altitude—dehydration, haemoconcentration, and vascular endothelial changes—can activate the coagulation cascade, potentially leading to myocardial infarction, cerebral venous thrombosis, and organ failure.
• Prompt diagnosis (MRI and thrombophilia testing) and early treatment (coronary artery bypass grafting, anticoagulation, and dialysis) were followed by full recovery in this patient.
• Even moderate altitude may meaningfully increase thrombotic risk in thrombophilia-prone individuals, supporting consideration of pre-ascent screening in those with a known predisposition.
• Protein C and S deficiency is a well-established risk factor for venous thromboembolism; its role in arterial and atherosclerotic coronary disease is considerably more contested. The coronary finding in this case may therefore represent coincidental pathology rather than a direct consequence of thrombophilia.
• Confirmatory testing was performed after the acute event had resolved. Postoperatively, protein C and protein S levels were 57% (normal >70%) and 55% (normal >65%), respectively. Genetic testing and family screening were not undertaken.

## Funding

This research has not received any specific grant from public, commercial, or non-profit sector agencies.

## Conflicts of interest

The authors have no conflicts of interest to declare.

## Ethical approval

This study was conducted after approval by the Ethics Committee of Ilam University of Medical Sciences (code of ethics: IR.MEDILAM.REC.1404.226).
